# Supplementary material for: Dynamic modulated arc therapy (DMAT): A time‐aware, modulation‐steered optimization framework for next‐generation radiotherapy delivery
Source: J Appl Clin Med Phys. 2026 Aug 24;27(9):e70764. doi: 10.1002/acm2.70764 (PMC13504227; doi:10.1002/acm2.70764)
Supplement: Supplementary file 1 [file ACM2-27-e70764-s001.docx]

**Table S-1.**

*Plan Score Cards used in this study*

Head and Neck with SIB (2.0/1.8/1.6 Gy x 35 fractions)

| **Structure** | **Parameter** | **Acceptable** | **Good** | **Ideal** | **Max Points** |
| --- | --- | --- | --- | --- | --- |
| **PTV70OPT** | V70Gy [%] | >=95% (15 pts) | >=97% (19 pts) | >=100% (20 pts) | 20 |
| **PTV63** | V63Gy [%] | >=95% (13 pts) | >=97% (16 pts) | >=100% (17 pts) | 17 |
| **PTV56** | V56Gy [%] | >=95% (13 pts) | >=97% (14.5 pts) | >=100% (15 pts) | 15 |
| **ParotidContra** | Dmean [Gy] | <=26Gy (0 pts) | <=18Gy (10 pts) | <=5Gy (15 pts) | 15 |
| **ParotidIpsi** | Dmean [Gy] | <=26Gy (7 pts) | <=18Gy (11 pts) | <=10Gy (12 pts) | 12 |
| **PTV70OPT** | D0.03cc [Gy] | <=77Gy (7 pts) | <=73.5Gy (9.5 pts) | <=71.3Gy (10 pts) | 10 |
| **PTV63-PTV70** | V66.15Gy [%] | <=75% (0 pts) | <=20% (7.5 pts); <=40% (6 pts) | <=10% (8 pts) | 8 |
| **PTV56-PTV63** | V58.8Gy [%] | <=80% (0 pts) | <=30% (7.5 pts);  <=50% (6 pts) | <=15% (8 pts) | 8 |
| **Lips** | Dmean [Gy] | <=20Gy (5 pts) | <=15Gy (6.5 pts) | <=10Gy (7 pts) | 7 |
| **Larynx-PTV** | Dmean [Gy] | <=20Gy (6 pts) | <=15Gy (6.75 pts) | <=10Gy (7 pts) | 7 |
| **SpinalCord_05** | D0.03cc [Gy] | <=50Gy (4 pts) | <=45Gy (6 pts) | <=25Gy (6.5 pts) | 6.5 |
| **OCavity-PTV** | Dmean [Gy] | <=32Gy (4.2 pts) | <=30Gy (4.5 pts) | <=15Gy (6 pts) | 6 |
| **Brain** | V10Gy [%] | <=10% (0 pts) | <=5% (4 pts) | <=0% (5 pts) | 5 |
| **PharConst-PTV** | Dmean [Gy] | <=50Gy (3 pts) | <=25Gy (4.5 pts) | <=10Gy (5 pts) | 5 |
| **Mandible-PTV** | V70Gy [%] | <=10% (0 pts) | <=6.5% (3.5 pts) | <=0% (5 pts) | 5 |
| **Posterior_Neck** | V35Gy [%] | <=50% (0 pts) | <=25% (4 pts) | <=0% (5 pts) | 5 |
| **BrainStem_03** | D0.03cc [Gy] | <=54Gy (2 pts) | <=50Gy (3 pts) | <=10Gy (4 pts) | 4 |
| **Esophagus** | Dmean [Gy] | <=30Gy (3 pts) <=56Gy (0 pts) | <=15Gy (3.75 pts) | <=5Gy (4 pts) | 4 |
| **BrachialPlexus_L** | D0.1cc [Gy] | <=66Gy (0 pts) | <=60Gy (3.5 pts) | <=50Gy (4 pts) | 4 |
| **BrachialPlexus_R** | D0.1cc [Gy] | <=66Gy (0 pts) | <=60Gy (3.5 pts) | <=50Gy (4 pts) | 4 |
| **Chiasm** | D0.03cc [Gy] | <=56Gy (0 pts) | <=54Gy (2.25 pts) | <=0Gy (3 pts) | 3 |
| **OpticNerve_L** | D0.03cc [Gy] | <=60Gy (0 pts) | <=56Gy (2 pts) | <=0Gy (3 pts) | 3 |
| **OpticNerve_R** | D0.03cc [Gy] | <=60Gy (0 pts) | <=56Gy (2 pts) | <=0Gy (3 pts) | 3 |
| **LacrimalGlands** | Dmean [Gy] | <=35Gy (0 pts) | <=17.5Gy (2 pts) | <=0Gy (3 pts) | 3 |
| **Cochlea_R** | V40Gy [%] | <=80% (0 pts) | <=50% (2 pts) | <=0% (3 pts) | 3 |
| **Cochlea_L** | V40Gy [%] | <=80% (0 pts) | <=50% (2 pts) | <=0% (3 pts) | 3 |
| **Esophagus** | D0.03cc [Gy] | <=70Gy (0 pts) | <=60Gy (2.5 pts) | <=56Gy (3 pts) | 3 |
| **Lens_R** | D0.03cc [Gy] | <=10Gy (0 pts) | <=5Gy (2 pts) | <=0Gy (2.5 pts) | 2.5 |
| **Lens_L** | D0.03cc [Gy] | <=10Gy (0 pts) | <=5Gy (2 pts) | <=0Gy (2.5 pts) | 2.5 |
| **Trachea** | Dmean [Gy] | <=65Gy (0 pts) | <=25Gy (1.75 pts) | <=0Gy (2.5 pts) | 2.5 |
| **SpinalCord_05** | V40Gy [%] | <=10% (0 pts) | <=5% (1.5 pts) | <=0% (2 pts) | 2 |
| **SpinalCord_05** | V30Gy [%] | <=45% (0 pts) | <=30% (1.5 pts) | <=0% (2 pts) | 2 |
| **Brain** | D0.03cc [Gy] | <=72Gy (0 pts) | <=70Gy (1.5 pts) | <=15Gy (2 pts) | 2 |
| **Eye_R** | D0.03cc [Gy] | <=50Gy (0 pts) | <=25Gy (1.5 pts) | <=0Gy (2 pts) | 2 |
| **Eye_R** | Dmean [Gy] | <=45Gy (0 pts) | <=20Gy (1.5 pts) | <=0Gy (2 pts) | 2 |
| **Eye_L** | D0.03cc [Gy] | <=50Gy (0 pts) | <=25Gy (1.5 pts) | <=0Gy (2 pts) | 2 |
| **Eye_L** | Dmean [Gy] | <=45Gy (0 pts) | <=20Gy (1.5 pts) | <=0Gy (2 pts) | 2 |
| **Mandible-PTV** | V60Gy [%] | <=35% (0 pts) | <=14% (1.75 pts) | <=0% (2 pts) | 2 |
| **Mandible-PTV** | V50Gy [%] | <=62% (0 pts) | <=31% (1.6 pts) | <=0% (2 pts) | 2 |
| **OCavity-PTV** | D0.03cc [Gy] | <=73.5Gy (0 pts) | <=60Gy (1.75 pts) | <=56Gy (2 pts) | 2 |
| **Thyroid-PTV** | Dmean [Gy] | <=40Gy (1.25 pts);  <=66Gy (0 pts) | <=25Gy (1.75 pts) | <=10Gy (2 pts) | 2 |
| **TMJoint** | D0.03cc [Gy] | <=75Gy (0 pts) | <=70Gy (1.5 pts) | <=0Gy (2 pts) | 2 |
| **RingPTV70** | D0.03cc [Gy] | <=77Gy (0 pts) | <=71.4Gy (1.75 pts) | <=70Gy (2 pts) | 2 |
| **RingPTV70** | V70Gy [cc] | <=15cc (0 pts) | <=5cc (1.75 pts) | <=0cc (2 pts) | 2 |
| **RingPTV63** | D0.03cc [Gy] | <=70Gy (0 pts) | <=64.26Gy (1.75 pts) | <=63Gy (2 pts) | 2 |
| **RingPTV63** | V63Gy [cc] | <=20cc (0 pts) | <=10cc (1.75 pts) | <=0cc (2 pts) | 2 |
| **RingPTV56** | D0.03cc [Gy] | <=65Gy (0 pts) | <=57.12Gy (1.75 pts) | <=56Gy (2 pts) | 2 |
| **RingPTV56** | V56Gy [cc] | <=50cc (0 pts) | <=25cc (1.75 pts) | <=0cc (2 pts) | 2 |
| **Lungs** | V20Gy [cc] | <=1000cc (0 pts) | <=500cc (1.5 pts) | <=0cc (2 pts) | 2 |
| **PTV70OPT** | D99% [Gy] | >=66.5Gy (0 pts) | >=67.2Gy (1 pt) | >=70Gy (1.5 pts) | 1.5 |
| **PTV63** | D99% [Gy] | >=59.85Gy (0 pts) | >=60.5Gy (1 pt) | >=63Gy (1.5 pts) | 1.5 |
| **PTV56** | D99% [Gy] | >=53.2Gy (0 pts) | >=53.75Gy (1 pt) | >=56Gy (1.5 pts) | 1.5 |
| **Pituitary** | Dmean [Gy] | <=45Gy (0.5 pts) | <=22.5Gy (0.8 pts) | <=0Gy (1 pt) | 1 |
| **Shoulders** | Dmean [Gy] | <=25Gy (0 pts) | <=10Gy (0.8 pts) | <=1Gy (1 pt) | 1 |

Prostate SBRT with SIB (10/7 Gy x 5 fractions)

| **Structure** | **Parameter** | **Acceptable** | **Good** | **Ideal** | **Max Points** |
| --- | --- | --- | --- | --- | --- |
| **PTV_P_P** | V33.25Gy [%] | >=90% (0 pts) | >=93% (30 pts) | >=95% (35 pts) | 35 |
| **Prostate_P1_P** | V35Gy [%] | >=90% (0 pts) | >=95% (18 pts) | >=100% (20 pts) | 20 |
| **GTVp** | V50Gy [%] | >=0% (0 pts) | >=97% (18 pts) | >=100% (20 pts) | 20 |
| **Rectum_P1_P** | V31.5Gy [cc] | <=2cc (0 pts) | <=1cc (13.5 pts) | <=0cc (15 pts) | 15 |
| **Bladder_P1_P** | V32.4Gy [cc] | <=5cc (0 pts) | <=3cc (9 pts) | <=0cc (15 pts) | 15 |
| **Rectum_P1_P** | D40% [Gy] | <=17.5Gy (0 pts) | <=13.2Gy (10 pts) | <=8.75Gy (13 pts) | 13 |
| **PTV_P_P** | D99.5% [Gy] | >=25.4Gy (0 pts) | >=28.5Gy (8 pts) | >=31.8Gy (10 pts) | 10 |
| **PTV_P_P** | ConformationNumber [] | Not specified | >=0.6 (0 pts) | >=1 (10 pts) | 10 |
| **Urethra_P_P** | D20% [Gy] | Not specified | <=38.5Gy (0 pts) | <=35Gy (10 pts) | 10 |
| **PTV_P_P** | V40Gy [%] | <=20% (0 pts) | <=15% (8 pts) | <=10% (10 pts) | 10 |
| **GTV Primary** | ConformationNumber [] | Not specified | >=0.5 (0 pts) | >=1 (10 pts) | 10 |
| **GTVp** | D0.1cc [Gy] | <=53Gy (0 pts) | <=52.5Gy (6 pts) | <=52Gy (8 pts) | 8 |
| **Rectum_P1_P** | V18Gy [cc] | <=50cc (0 pts) | <=25cc (6 pts) | <=12.5cc (8 pts) | 8 |
| **Bladder_P1_P** | V18Gy [cc] | <=50cc (0 pts) | <=25cc (6 pts) | <=12.5cc (8 pts) | 8 |
| **Bladder_P1_P** | V36Gy [cc] | Not specified | <=5cc (0 pts) | <=0cc (5 pts) | 5 |
| **Bowel_P_P** | D1cc [Gy] | Not specified | <=26.3Gy (0 pts) | <=0Gy (5 pts) | 5 |
| **penile bulb_P_P** | D0.1cc [Gy] | Not specified | <=29.5Gy (0 pts) | <=10Gy (3 pts) | 3 |
| **Neurovascular B1** | D50% [Gy] | Not specified | <=37.5Gy (0 pts) | <=35Gy (3 pts) | 3 |
| **Right Femoral H1** | D0.03cc [Gy] | <=24Gy (0 pts) | <=12.25Gy (2.7 pts) | <=8.75Gy (3 pts) | 3 |
| **Left Femoral He1** | D0.03cc [Gy] | <=24Gy (0 pts) | <=12.25Gy (2.7 pts) | <=8.75Gy (3 pts) | 3 |
| **Skin_P_P** | D0.03cc [Gy] | Not specified | <=26.25Gy (0 pts) | <=8.75Gy (3 pts) | 3 |
| **Testes_P_P** | D0.03cc [Gy] | Not specified | <=1.75Gy (0 pts) | <=0Gy (3 pts) | 3 |

Lung SBRT ( 10Gy x 5 fractions)

| **Structure** | **Parameter** | **Acceptable** | **Good** | **Ideal** | **Max Points** |
| --- | --- | --- | --- | --- | --- |
| **PTV** | V50Gy [%] | >=90% (0 pts) | >=95% (19 pts) | >=100% (20 pts) | 20 |
| **ITV** | V50Gy [%] | Not specified | >=98% (0 pts) | >=100% (20 pts) | 20 |
| **BODY** | V25Gy [cc] | <=70cc (0 pts) | <=65cc (15 pts) | <=57.4cc (20 pts) | 20 |
| **Lungs-ITV** | V20Gy [%] | <=15% (0 pts) | <=10% (11 pts) | <=5% (12 pts) | 12 |
| **HEART** | Dmean [Gy] | Not specified | <=10Gy (0 pts) | <=3Gy (12 pts) | 12 |
| **PTV_SUB_ITV** | D0.03cc [Gy] | <=75Gy (0 pts) | <=65Gy (9 pts) | <=57.5Gy (10 pts) | 10 |
| **ITV** | D0.03cc [Gy] | <=83Gy (0 pts) | <=75Gy (9 pts) | <=60Gy (10 pts) | 10 |
| **Lungs-ITV** | V13.5Gy [%] | <=7% (0 pts) | <=3% (9.5 pts) | <=1% (10 pts) | 10 |
| **ESOPHAGUS** | D0.03cc [Gy] | Not specified | <=38Gy (0 pts) | <=10Gy (10 pts) | 10 |
| **SKIN** | D0.03cc [Gy] | <=39.5Gy (0 pts) | <=32Gy (5 pts); <=38.5Gy (2.5 pts) | <=15Gy (6 pts) | 6 |
| **SKIN** | D10cc [Gy] | <=36.5Gy (0 pts) | <=30Gy (5 pts) | <=5Gy (6 pts) | 6 |
| **PTV** | V45Gy [%] | Not specified | >=99% (0 pts) | >=100% (5 pts) | 5 |
| **PTV** | D99.5% [Gy] | Not specified | >=45Gy (0 pts) | >=47.5Gy (5 pts) | 5 |
| **BODY** | V50Gy [cc] | <=18.01cc (0 pts) | <=18cc (4 pts) | <=14.4cc (5 pts) | 5 |
| **RING_2CM** | MaxDose [Gy] | <=29.01Gy (0 pts) | <=29Gy (4 pts) | <=25Gy (5 pts) | 5 |
| **HEART** | D0.03cc [Gy] | <=38Gy (0 pts) | <=20Gy (4.5 pts) | <=15Gy (5 pts) | 5 |
| **Bronchial tree right** | D0.03cc [Gy] | <=52.5Gy (0 pts) | <=50Gy (4 pts) | <=40Gy (5 pts) | 5 |
| **Ribs right** | D0.03cc [Gy] | <=57Gy (0 pts) | <=52.5Gy (4.75 pts) | <=43Gy (5 pts) | 5 |
| **Ribs right** | V35Gy [cc] | Not specified | <=8cc (0 pts) | <=1cc (5 pts) | 5 |
| **Chest wall right** | V30Gy [cc] | <=70cc (0 pts) | <=30cc (4 pts) | <=0cc (5 pts) | 5 |
| **Lungs-ITV** | Dmean [Gy] | <=8Gy (0 pts) | <=4Gy (3 pts) | <=0Gy (4 pts) | 4 |
| **SPINAL_CORD** | D0.03cc [Gy] | <=30Gy (0 pts) | <=28Gy (2.5 pts) | <=22Gy (3 pts) | 3 |
| **ESOPHAGUS** | V27.5Gy [cc] | Not specified | <=5cc (0 pts) | <=3cc (3 pts) | 3 |
| **BRACHIAL_PLEXUS** | D0.03cc [Gy] | <=32.5Gy (0 pts) | <=32Gy (2.5 pts) | <=30.5Gy (3 pts) | 3 |
| **BRACHIAL_PLEXUS** | D3Gy [cc] | Not specified | <=30cc (0 pts) | <=27cc (3 pts) | 3 |
| **Bronchial tree right** | D4cc [Gy] | Not specified | <=45Gy (0 pts) | <=18Gy (3 pts) | 3 |
| **STOMACH** | D0.03cc [Gy] | <=35Gy (0 pts) | <=32Gy (2 pts) | <=15Gy (3 pts) | 3 |
| **LIVER** | D700Gy [cc] | <=21.5cc (0 pts) | <=21cc (0.5 pts) | <=15cc (3 pts) | 3 |
| **SPINAL_CORD** | V13.5Gy [cc] | Not specified | <=1.2cc (0 pts) | <=0.5cc (2 pts) | 2 |
| **HEART** | V32Gy [cc] | Not specified | <=15cc (0 pts) | <=1cc (2 pts) | 2 |
| **GREAT_VESSELS** | V47Gy [cc] | Not specified | <=10cc (0 pts) | <=1cc (2 pts) | 2 |
| **GREAT_VESSELS** | D0.03cc [Gy] | Not specified | <=53Gy (0 pts) | <=50Gy (2 pts) | 2 |
| **TRACHEA** | V16.5Gy [cc] | Not specified | <=4cc (0 pts) | <=1cc (2 pts) | 2 |
| **TRACHEA** | D0.03cc [Gy] | Not specified | <=52.5Gy (0 pts) | <=50Gy (2 pts) | 2 |
| **Chest wall right** | D0.03cc [Gy] | Not specified | <=52.5Gy (0 pts) | <=50Gy (2 pts) | 2 |
| **STOMACH** | V18Gy [cc] | Not specified | <=10cc (0 pts) | <=5cc (2 pts) | 2 |
| **STOMACH** | V26.5Gy [cc] | Not specified | <=5cc (0 pts) | <=0cc (2 pts) | 2 |
